# *Kcnab1* Is Expressed in Subplate Neurons With Unilateral Long-Range Inter-Areal Projections

**DOI:** 10.3389/fnana.2019.00039

**Published:** 2019-05-03

**Authors:** Sheena Yin Xin Tiong, Yuichiro Oka, Tatsuya Sasaki, Manabu Taniguchi, Miyuki Doi, Hisanori Akiyama, Makoto Sato

**Affiliations:** ^1^Department of Anatomy and Neuroscience, Graduate School of Medicine, Osaka University, Osaka, Japan; ^2^Division of Developmental Neuroscience, Department of Child Development, United Graduate School of Child Development, Osaka University, Kanazawa University, Hamamatsu University School of Medicine, Chiba University and University of Fukui, Osaka, Japan; ^3^Institute of Biological Sciences, Faculty of Science, University of Malaya, Kuala Lumpur, Malaysia; ^4^Division of Cell Biology and Neuroscience, Department of Morphological and Physiological Sciences, Faculty of Medical Sciences, University of Fukui, Fukui, Japan; ^5^Research Center for Child Mental Development, University of Fukui, Fukui, Japan

**Keywords:** subplate, axon projection, *Kcnab1*, cerebral cortex, potassium channel, corticocortical projection, neuronal circuit, FluoroGold

## Abstract

Subplate (SP) neurons are among the earliest-born neurons in the cerebral cortex and heterogeneous in terms of gene expression. SP neurons consist mainly of projection neurons, which begin to extend their axons to specific target areas very early during development. However, the relationships between axon projection and gene expression patterns of the SP neurons, and their remnant layer 6b (L6b) neurons, are largely unknown. In this study, we analyzed the corticocortical projections of L6b/SP neurons in the mouse cortex and searched for a marker gene expressed in L6b/SP neurons that have ipsilateral inter-areal projections. Retrograde tracing experiments demonstrated that L6b/SP neurons in the primary somatosensory cortex (S1) projected to the primary motor cortex (M1) within the same cortical hemisphere at postnatal day (PD) 2 but did not show any callosal projection. This unilateral projection pattern persisted into adulthood. Our microarray analysis identified the gene encoding a β subunit of voltage-gated potassium channel (*Kcnab1*) as being expressed in L6b/SP. Double labeling with retrograde tracing and *in situ* hybridization demonstrated that *Kcnab1* was expressed in the unilaterally-projecting neurons in L6b/SP. Embryonic expression was specifically detected in the SP as early as embryonic day (E) 14.5, shortly after the emergence of SP. Double immunostaining experiments revealed different degrees of co-expression of the protein product Kvβ1 with L6b/SP markers Ctgf (88%), Cplx3 (79%), and Nurr1 (58%), suggesting molecular subdivision of unilaterally-projecting L6b/SP neurons. In addition to expression in L6b/SP, scattered expression of *Kcnab1* was observed during postnatal stages without layer specificity. Among splicing variants with three alternative first exons, the variant 1.1 explained all the cortical expression mentioned in this study. Together, our data suggest that L6b/SP neurons have corticocortical projections and *Kcnab1* expression defines a subpopulation of L6b/SP neurons with a unilateral inter-areal projection.

## Introduction

The subplate (SP) is known to be a transient layer that contributes substantially to early cortical development, whereas most of its constituent neurons gradually disappear when the cortical layers start to emerge and only a small portion of SP neurons remain in layer 6b (L6b) as development proceeds (Reep and Goodwin, 1988; Chun and Shatz, 1989; Bayer and Altman, 1990; Kostovic and Rakic, 1990; Woo et al., 1991; Allendoerfer and Shatz, 1994; Valverde et al., 1995; Clancy and Cauller, 1999; Reep, 2000; Robertson et al., 2000; Hanganu et al., 2002; Kanold, 2009; Hoerder-Suabedissen and Molnár, 2015). The L6b/SP neurons that remain into adulthood regulate cortical layer formation and modulate information flow into and out of the cortex (Kanold and Shatz, 2006; Friedlander and Torres-Reveron, 2009; Kanold and Luhmann, 2010). In rodents amongst other mammalian species, L6b/SP neurons are morphologically heterogeneous (Kostovic and Rakic, 1990; Valverde et al., 1995; Hanganu et al., 2002; Andjelic et al., 2009; Chen et al., 2009; Hoerder-Suabedissen and Molnár, 2012; Marx and Feldmeyer, 2013; Marx et al., 2017) and diverse in gene expression (Hoerder-Suabedissen et al., 2009; McKellar and Shatz, 2009; Osheroff and Hatten, 2009; Belgard et al., 2011; Oeschger et al., 2012; Hoerder-Suabedissen and Molnár, 2013; Tasic et al., 2016). Electrophysiological studies also revealed distinct intrinsic properties of SP neurons that most likely play an important role in shaping cortical networks during development (Hanganu et al., 2002; Dupont et al., 2006; Luhmann et al., 2009, 2018; Kanold and Luhmann, 2010; Yang et al., 2016; Deng et al., 2017).

SP neurons consist largely of projection neurons, and their axons begin to form projections very early on during development (McConnell et al., 1989; De Carlos and O’Leary, 1992; Clascá et al., 1995; Molnár et al., 1998; Pedraza et al., 2014). SP projection neurons extend their axons to specific cortical and subcortical sites (Usrey and Fitzpatrick, 1996; Zhang and Deschênes, 1997; Kanold and Luhmann, 2010; Hoerder-Suabedissen and Molnár, 2012; Hoerder-Suabedissen et al., 2018). Intracortical axonal projections from the SP target layers 1 and 4 above the SP and also distant cortical regions (Clancy and Cauller, 1999; Piñon et al., 2009; Zhao et al., 2009; Hoerder-Suabedissen and Molnár, 2012; Hoerder-Suabedissen et al., 2018). Axon collaterals of SP neurons project both ipsilaterally and contralaterally within the cortex, although only very few of the latter were found in rodents (De Carlos and O’Leary, 1992; Allendoerfer and Shatz, 1994; Ozaki and Wahlsten, 1998; Del Río et al., 2000; Hanganu et al., 2002; Kanold et al., 2003; Kanold and Shatz, 2006; Hoerder-Suabedissen and Molnár, 2012). Similar intracortical axon projection patterns have been reported in other mammalian species including cats and ferrets (McConnell et al., 1989; Antonini and Shatz, 1990; Friauf et al., 1990; Friauf and Shatz, 1991; Finney et al., 1998).

Molecular characterization of SP neurons at different developmental stages has identified SP-specific genes that are mostly temporal, which indicates the possible changing role of SP neurons underlying functional circuitry development and refinement (Hoerder-Suabedissen et al., 2009, 2013; Oeschger et al., 2012; Hoerder-Suabedissen and Molnár, 2013; Sorensen et al., 2015; Viswanathan et al., 2017). Several studies have shown that deletion of SP resulted in disrupted cortical plate organization and developmental deficits in brain functions (Ghosh et al., 1990; Ghosh and Shatz, 1993; Lein et al., 1999; Kanold et al., 2003; Kanold and Shatz, 2006; Magnani et al., 2012; Tolner et al., 2012). Thus, expression of SP-specific genes at a critical period during development almost certainly underlies the fine orchestration of SP axon projection pattern and regulates cortical maturation. However, the relationship between molecular identity and axon projection target specificity remains largely elusive.

Our study is an effort to characterize axon projection pattern in the mouse L6b/SP, and to correlate it to the specific gene expression pattern of these neurons. This is crucial to understand the diversity of L6b/SP neurons and to enable functional manipulation of the discrete classes to clarify the distinct role of these neurons during cortex maturation. We set out to examine the axonal projection target of L6b/SP neurons and whether this is consistent throughout development. We studied axonal projection from L6b/SP of the primary somatosensory cortex (S1) to the primary motor cortex (M1) since revealing molecular identity and development of this projection would lead to better understanding of a parallel circuit that it forms with the projections from the other layers (Mitchell and Macklis, 2005). We found a proportion of L6b/SP neurons that project unilaterally from S1 to M1 can be molecularly identified by a specific splice variant of the voltage-gated potassium channel *Kcnab1*. This discrete group of neurons was present at different stages of development, although the expression pattern of *Kcnab1* changed as the brain matured. There was also a considerable overlap between the Kvβ1-positive neurons in L6b/SP with known L6b/SP molecular markers. Overall, our results suggested that this molecularly distinctive group of neurons is most likely a L6b/SP subpopulation that survives as the cortex develops and may underlie inter-areal circuit establishment in the cortex.

## Materials and Methods

### Animals

Wild-type ICR mice were used in this study, and the animals were purchased from SLC Japan and housed under standard conditions with food and water *ad libitum* and maintained on a 12-h light/dark cycle. Embryonic day (E) 0.5 was defined as 12:00 noon on the day when the vaginal plug was found. During all surgical protocols, animals were deeply anesthetized by intraperitoneal injection of a combination anesthetic (MMB: 0.3 mg/kg of medetomidine, 4.0 mg/kg of midazolam, and 5.0 mg/kg of butorphanol) and intracardially perfused with ice-cold phosphate buffered saline (PBS) followed by 4% paraformaldehyde (PFA). Whole brains were carefully dissected after perfusion and post-fixed in 4% PFA overnight at 4°C, then transferred to 30% sucrose solution (≥24 h or until brain sinks to the bottom of the tube at 4°C). In the case of mouse embryos, the embryos were collected from pregnant dams (wild-type ICR) and the brains were immediately dissected from the embryos and post-fixed in 4% PFA overnight at 4°C, then transferred to 30% sucrose solution. The dams were sacrificed with cervical dislocation after administration of five doses of MMB. All animal experiments were approved by the Animal Research Committee of University of Fukui and the Animal Experimentation Committee of Osaka University, and performed in accordance with the Regulations for Animal Research at the University of Fukui and the Regulations on Animal Experimentation at Osaka University.

### Retrograde Labeling and Tissue Processing

Postnatal day (PD) 2 and 3-week-old (PW3) mice were used in the retrograde tracing experiments. PW3 mice were anesthetized and fixed on a stereotaxic frame, and tracer was injected into the cortex according to coordinates determined based on the mouse brain atlas (Paxinos and Franklin, 2008). Tracer used in this study was 2% Fluoro-Gold (Fluorochrome) in distilled water; 0.5% Cholera Toxin Subunit B conjugated with AlexaFluor 488 (Thermo Fisher Scientific) in PBS; or 2% Green RetroBeads^TM^ IX (Lumaflour) in PBS, and all tracer mixtures were added with 0.1 μg/ml Fast Green. For all injection sites, the anterior/posterior (AP) coordinates were referenced from Bregma, the medial/lateral (ML) coordinates were the distance from the midline at Bregma, and the dorsal/ventral (DV) coordinates were measured from the pial surface of the brain. All measurement units were in mm and are referred to in the following description as [anterior/posterior (AP), medial/lateral (ML), dorsal/ventral (DV)]. Three and six injection sites were selected for both M1 and S1, respectively. For injection into M1, the stereotaxic coordinates were (0.7, 1.0, 0.8), (1.2, 1.2, 0.8), and (1.5, 1.5, 0.8); whereas the stereotaxic coordinates for injection into S1 were (0.0, 3.0/3.5, 0.5), (−0.50, 3.0/2.5, 0.5), and (−1.5, 3.0/2.5, 0.5). Each animal received pressure injection (approximately 40 nl/site) of a tracer delivered *via* a glass needle (pulled and broken at the tip with forceps to make an opening with a diameter of 40–60 μm) attached to a Hamilton syringe. After 4 days’ survival, animals were transcardially perfused and the brains were processed as mentioned above. Images of the whole FG-injected brains were taken with a fluorescence stereomicroscope (MZ 10F, Leica) with a filter set ET UV LP (Leica), and only the brains that appeared to have sufficient injection were subsequently sectioned. The selected brains were embedded in O.C.T. compound (Sakura) and stored at −80°C, then cut into 14–16 μm sections on a coronal plane using a freezing microtome (Leica model CM3050S). Sections were imaged with BZ-X700 (Keyence) using an appropriate filter cube for FG (ET DAPI/FluoroGold, Chroma Technology Co., Bellows Falls, VT, USA).

As for mice at PD2, the animals were anesthetized by hypothermia and tracer was injected by using a Picospritzer^®^ II microinjector (Parker Hannifin, Cleveland, OH, USA). The injection targets (M1 and S1) were estimated based on parallel experiments whereby the injected mouse was raised to adulthood and injection sites in M1 and S1 were traced to confirm the point of injection. Briefly, injection in M1 and S1 were estimated as referral points from the midline (x) and the most rostral edge of the cortex (y), whereby M1 was x: 1.5 mm, y: 2.0 mm; and S1 was x: 2.5–2.8 mm and y: 3.0–3.5 mm. The mice were let to recover on heated pads and returned to the dams for survival. The mice were perfused 2 days post-injection at PD4 and the brains were processed and imaged as mentioned above.

### Microarray Screening

The genes preferentially expressed in association neurons (projecting from S1 to ipsilateral M1) compared to those in callosal neurons [projecting from S1 to contralateral S1 (cS1)] were identified with DNA microarray screening. In brief, cortical layer 2/3 neurons were transfected with pCAGGS-tdTomato by performing *in utero* electroporation at E15.5, and the mice that had tdTomato labeling of cell bodies in S1 were used in the subsequent procedure. The retrograde tracer Green Retrobeads^TM^ IX was injected at PD21 into either M1 or cS1 with the help of red fluorescence of tdTomato in these areas (in axon terminals projecting from S1). Mice were transcardially perfused with ice-cold PBS and the brains were dissected out, embedded in O.C.T. compound, and frozen in powdered dry ice. Fresh-frozen sections were cut at 10 μm, thaw-mounted onto glass slides covered with polyphenylene sulfide membrane and air-dried immediately. The labeled association neurons in layers 2/3, 5, and 6b, and labeled callosal neurons in layers 2/3 and 5 were collected from S1 (more than 1,000 cells each) using a laser-captured microdissection system (AS-LMD, Leica). Total RNAs were prepared using NucleoSpin RNA XS kit (Macherey-Nagel). cDNAs were synthesized, amplified, Biotin-labeled and fragmented using Ovation^TM^ Pico WTA System, WT-Ovation^TM^ Exon Module, and Encore^TM^ Biotin Module (NuGEN). Labeled cDNAs were hybridized to the GeneChip Mouse Gene 1.0 ST Array (Affymetrix, Santa Clara, CA, USA). Hybridization, washing and scanning were performed with a GeneChip 3,000 7G system (Affymetrix, Santa Clara, CA, USA). Raw signals were subjected to Log-transformation, global normalization, and centering in order to obtain processed signals for cross-sample comparison. The candidate genes preferentially expressed in L6b were selected using Subio Platform (version 1.14, Subio). The microarray data were deposited at Gene Expression Omnibus (GEO) under the accession number GSE123351.

### Probe Generation and *In situ* Hybridization Histochemistry (ISHH)

cDNA fragments of *Kcnab1* and *Ctgf* were PCR-amplified from mouse brain cDNA with the following primer pairs. *Kcnab1*-fwd.: 5′ GAAATGGGGTGCCAGAAA 3′; rev.: 5′ ATTGTACAGGGCCAGGCA 3′; *Ctgf*-fwd.: 5′ AGAGTGGAGCGCCTGTTCTA 3′; and rev.: 5′ ACTGGCAGAGTGGTGGTTCT 3′. In order to examine the expression pattern of *Kcnab1* splicing variants *Kcnab1.1*, *Kcnab1.2*, and *Kcnab1.3* in the mouse cortex, *in situ* probes were generated to specifically recognize each of the subunits. The primer sets used were as follows: *Kcnab1.1*-fwd.: 5′ CAGCCGAGATCACAGCCTG… 3′; rev.: 5′ CTGCTTTGCGGTGGACTCTT… 3′; *Kcnab1.2*-fwd.: 5′ ATAAACCTGCCTGTGCAGA… 3′; rev.: 5′ CATGCCTGTCTTTGCCTTG… 3′; *Kcnab1.3*-fwd.: 5′ AGGCAGATAGGAACTTCCAG… 3′; rev.: 5′ GCTCGCAGAGCTTTAGGT… 3′. Amplified fragments were cloned into pGEM-T vector (Promega). *In vitro* transcription of cRNA probes was performed with T7 or SP6 RNA polymerase (Roche) using the template plasmids linearized with an appropriate restriction enzyme and RNA DIG labeling mix (Roche) according to the manufacturer’s instructions.

*In situ* hybridization histochemistry (ISHH) was performed as described before (Yagi et al., 2016) using cryosections (14–16 μm) prepared from mice at E12.5, E14.5, E16.5, and E18.5; and PD3, PD4, PD7, PD21, and PD25 as mentioned above. The cryosections were air dried for 1 h and fixed in 4% PFA in PBS for 10 min at room temperature. The sections were then incubated in 0.2 M HCl for 10 mins, followed by permeabilization with Proteinase K (7.9 μg/ml; Roche) digestion for 10 min at 37°C. Concentration and treatment duration of Proteinase K were halved for the brain samples younger than PD4. Next, the sections were treated with acetic anhydride in 0.1 M triethanolamine for 10 min. The slides were rinsed with PBS in between each step. Finally, the sections were transferred to 5× saline sodium citrate (SSC) for 10 min or longer. Hybridization was carried out with the generated probes in hybridization buffer (50% formamide, 5× SSC, 200 μg/ml yeast tRNA) overnight for at least 16 h at 55°C. High-stringency washes were carried out in the following steps: 5× SSC, 20 min at room temperature; 2× SSC, 20 min at 65°C; two washes with 0.2× SSC, 20 min at 65°C and lastly the slides were transferred to PBS at room temperature. Detection of specific hybridization was performed using anti-Digoxigenin coupled with alkaline phosphatase, and subsequently visualized using nitro blue tetrazolium chloride/5-bromo-4-chloro-3-indolyl-phosphate (NBT/BCIP). Sense probes were used as negative controls and no signals were observed with the sense probes. Bright field images of the stained sections were taken with BZ-X700.

### Immunohistochemistry

Brain cryosections were prepared from adult mice (PW8–PW24) and the sections were processed accordingly for double immunofluorescence labeling. After air-drying for an hour at room temperature, the sections were treated with Tris EDTA buffer (pH 8.5) for 1 min at 105°C in the autoclave or for 30 min at 85°C in the water bath. This was followed by short rinses (5 min, three rinses) in PBS before blocking the sections with 5% normal donkey serum, 0.1% TritonX-100 in PBS. The sections were then incubated in primary antibodies (diluted in blocking solution) overnight at 4°C. The primary antibodies used in this study were as follows: CaMKIIα (1:100; rabbit; GeneTex, Irvine, CA, USA), Cplx3 (1:500; rabbit; SYSY), Ctgf (1:200; goat; Santa Cruz, CA, USA), Kvβ1 (1:200; mouse; Santa Cruz), and Nurr1 (1:200; goat; R&D). This was followed by incubation in species-specific fluorescent secondary antibodies (Donkey anti-mouse IgG Alexa Fluor 568 and either Donkey anti-rabbit IgG Alexa Fluor 488 or Donkey anti-goat IgG Alexa Fluor 488, all from Thermo Fisher Scientific, Waltham, MA, USA) for 4–6 h at 4°C. All secondary antibodies were diluted at 1:200 in PBS. Nuclear staining was performed using 4′,6-diamidino-2-phenylindole (DAPI). Fluorescence signals were imaged with a laser scanning confocal microscope (LSM 880 with Airyscan, Zeiss).

Immunohistochemistry against Fluoro-Gold was combined with ISHH. After detection of ISHH signals, sections were incubated with anti-Fluoro-Gold (FG; 1:500; rabbit; Millipore, MA, USA) overnight at 4°C. Immunoperoxidase labeling was performed using a Vectastain Elite ABC Kit (Vector) and a DAB detection kit (Vector) was used for detection, according to the manufacturer’s protocol. Bright field images of the stained sections were taken with BZ-X700.

### Splice Variant Identification

Mouse homologues for exons 1.2 and 1.3 of the human *Kcnab1* gene were identified by T-BLAST-N search in Ensembl genome database (GRCm38.p6^1^) with the human sequences as queries. The genomic regions encompassing the hit sequences and exon 2 were then subjected to exon/intron prediction with GeneWise^2^. The identified sequences were deposited at the DNA Data Bank of Japan (DDBJ) under the accession numbers LC437679 for exon 1.2 and LC437680 for exon 1.3.

### Cell Quantification

For analysis of retrogradely labeled L6b/SP neurons that expressed *Kcnab1*, 3 to 7 S1-containing sections per animal × three animals were used for quantification. The bright field images were adjusted for brightness and contrast using Adobe Photoshop CS3 (Adobe). Cell quantification was carried out using the Cell Counter plugin for ImageJ software (National Institutes of Health, Bethesda, MD, USA). Cells within a 4-cell-height from the border between the cortex and the white matter were considered as L6b and included in quantification. While the NBT/BCIP signals were usually devoid of nucleus, DAB tended to stain the entire cell body. Thus, double-positive cells were defined as cells that had DAB signal surrounded by NBT/BCIP signal. Cells without a clear nucleus were not included in quantification. Cells that were too densely stained with NBT/BCIP and/or DAB were excluded. The cell numbers counted are summarized in Supplementary Table S1.

For co-localization analysis for Kvβ1 and L6b markers, three S1-containing sections per animal × three animals were used for quantification. The fluorescence images were adjusted for brightness and contrast using Adobe Photoshop CS3. Cell quantification was carried out using the Cell Counter plugin for ImageJ software. Cells within a 4-cell-height from the border between the cortex and the white matter were considered as L6b and included in quantification. Only the cells with DAPI signals were included in quantification. DAPI-positive cells were also counted to estimate the total cell number in each counted area. Co-localization analysis for Kvβ1 and CaMKIIα was performed in the same manner using two sections per animal × four animals. The cell numbers counted are summarized in Supplementary Table S2.

### Analysis of the Public RNAseq Data in the Gene Expression Omnibus (GEO) Database

The TPM (Transcripts per million) data for *Kcnab1*, *Ctgf*, *Cplx3*, and *Nurr1* (*Nr4a2*) genes across 1809 individual cells isolated from the mouse primary visual cortex (V1) were obtained from the GEO^3^ (accession, GSE 71585; Tasic et al., 2016). Genes were considered as being expressed in a cell when the TPM value in the cell was 10 or larger. The percentage of cells expressing *Kcnab1* at two different expression levels (TPM ≥ 10 and TPM ≥ 100) was calculated for each of the eight broad types of cortical cells (astrocytes, endothelial cells, GABAergic neurons, glutamatergic neurons, microglia, oligodendrocytes, oligodendrocyte precursor cells, and unclassified cells). The percentages were also calculated for glutamatergic neurons in different layers and seven classes of GABAergic neurons by combining the numbers of cells for primary cell types in each layer or class. The percentages of cells expressing each of the four genes were calculated for two primary cell types for L6b (Rgs12 and Sepinb11). To specifically examine if *Kcnab1* is expressed in GABAergic neurons in L6b, the percentages of cells expressing *Kcnab1* among 41 cells (containing 33 glutamatergic neurons and eight GABAergic neurons) dissected from L6b of V1 were also calculated.

Similar analyses were performed using other RNAseq data for the four genes across 3,005 individual cells isolated from mouse S1 and hippocampal CA1 (Zeisel et al., 2015). The annotated expression data (equivalent to the raw data in GEO with the accession GSE60361 except for the cell type annotations) were obtained from the website of Linnarsson lab^4^. The percentages of *Kcnab1*-expressing cells (expression score ≥ 1) were calculated for Level-1 classes of cell types and for Level-2 classes of interneurons and pyramidal neurons. Those of cells expressing each of the four genes were calculated for L6b cells.

## Results

### Layer 6b/SP Contained Corticocortical Projecting Neurons

**Figure 1:**
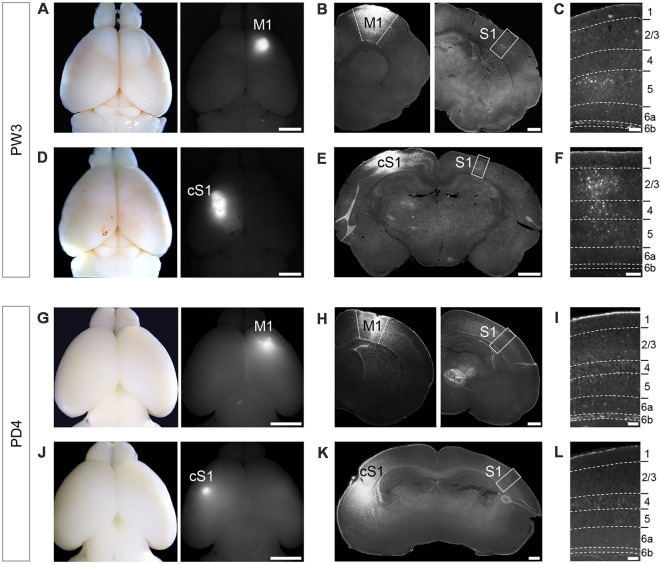
Retrograde tracer injection in M1 of PW3 mouse brain labeled unilaterally-projecting Layer 6b (L6b)/SP neurons in primary somatosensory cortex (S1). **(A,D)** Whole mount brain images in a bright field (left) and a fluorescent (right) views showing Fluoro-Gold (FG) injections into primary motor cortex (M1; **A**) and contralateral S1 (cS1; **D)** at PW3. Scale bars, 2 mm. **(B)** Coronal sections of the brain shown in **(A)** at the level of M1 (left) and S1 (right). Scale bar, 500 μm. **(C)** Higher magnification view of the boxed area in **(B)**. FG-labeled neurons were detected in L6b, as well as other layers. Scale bar, 100 μm. **(E)** A coronal section of the brain shown in **(D)** at the level of S1. Scale bar, 500 μm. **(F)** Higher magnification view of the boxed area in **(E)**. Scale bar, 100 μm. **(G,J)** Whole mount brain images in a bright field (left) and a fluorescent (right) views showing FG injection into M1 **(G)** and cS1 **(J)** at PD2. Scale bars, 2 mm. **(H)** Coronal sections of the brain shown in **(G)** at the level of M1 (left) and S1 (right). Scale bar, 500 μm.** (I)** Higher magnification views of the boxed area in **(H)**. FG-labeled neurons were detected in L6b. Scale bar, 100 μm.** (K)** A coronal section of the brain shown in **(J)** at the level of S1. Scale bar, 500 μm. **(L)** Higher magnification views of the boxed area in **(K)**. Scale bars, 100 μm. Dashed lines in **(C)**, **(F)**, **(I)**, and **(L)** indicate borders of cortical layers.

Discrete neuronal populations occupy each cortical layer and the laminar location of cell bodies commonly corresponds to specific axon projection targets. In order to determine whether neurons in the S1 area project over a long distance to the M1 area or form callosal projections, we injected retrograde tracer FG in M1 and cS1 of mice at age PW3 (Figures 1A,D). This was followed by a survival time of 4 days to allow the tracer to be retrogradely transported. Then we examined the distribution of retrogradely-labeled neurons in S1. We observed FG-labeled neurons in L6b in addition to some in layers 2/3 and 5, when tracer was injected into M1 (Figures 1B,C), consistent with a previous report (Mitchell and Macklis, 2005). When FG was injected in S1 in the other hemisphere under the same condition, there was no labeled neuron in L6b, although FG-labeled neurons were obvious in layers 2/3 to 5 (Figures 1E,F). The retrograde labeling experiments showed the same results when repeated with two other tracers; fluorescence conjugated-cholera toxin B (CTB) and Green RetroBeads^TM^ (data not shown).

We next asked if this projection pattern is consistent throughout postnatal development. When FG was injected at PD2, we found FG-labeled SP neurons at PD4 for tracing from M1 (Figures 1G–I), but not for tracing from cS1 (Figures 1J–L), similar to the results in L6b neurons in PW3 animals. Taken together, L6b/SP neurons in the S1 area projected their axons unilaterally to the M1 area.

### *Kcnab1* Was Identified as a Candidate Gene Expressed in L6b/SP Association Neurons

**Figure 2:**
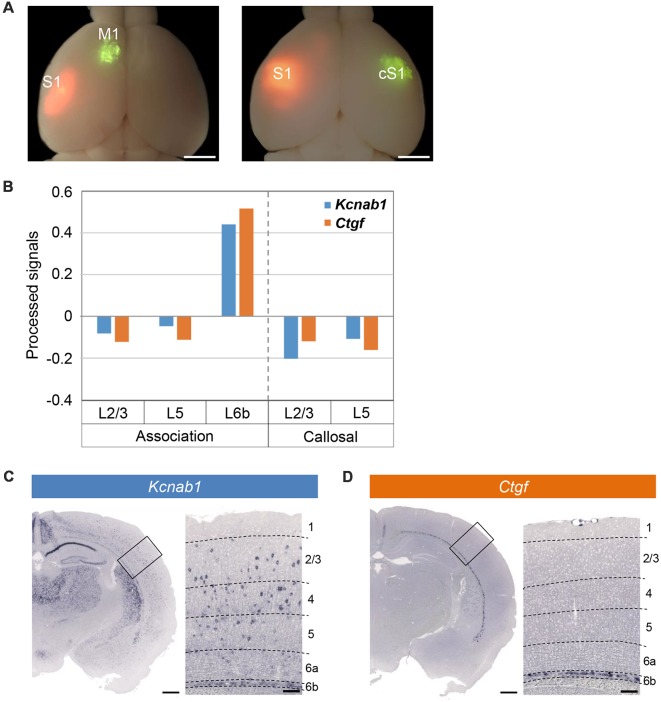
Microarray screening identified *Kcnab1* and *Ctgf* as expressed in L6b. **(A)** Green RetroBeads^TM^ was injected into M1 (left panel) or cS1 (right panel) of the brains at PD21 that had been electroporated with pCAGGS-tdTomato at E15.5. Green and red fluorescent images were overlaid onto the bright field image. Scale bars, 2 mm.** (B)** Relative expression levels of *Kcnab1* (blue) and *Ctgf* (orange) among association neurons in L2/3, L5, and L6b and callosal neurons in L2/3 and L5 compared by microarray analysis. **(C,D)** *In situ* hybridization histochemistry (ISHH) for *Kcnab1* **(C)** and *Ctgf* **(D)** was carried out on coronal sections of mouse brain at PD21. Higher magnification view of the boxed area is shown on the right of each panel. Dashed lines in these high magnification images indicate borders of cortical layers. Scale bars, 500 μm and 100 μm for low and high magnifications, respectively.

In our DNA microarray screening for the genes preferentially expressed in association neurons (projecting from S1 to M1) and those in callosal neurons (projecting from S1 to cS1; Figure 2A), we found many genes preferentially expressed in the association neurons in L6b/SP. We selected 100 candidate genes with the highest enrichment in L6b/SP for shortlisting by ISHH. These included a gene encoding a β subunit of voltage-dependent potassium channel, *Kcnab1*, as well as known L6b/SP markers including *Ctgf* (Figure 2B). ISHH analysis with coronal sections of the PD21 cortex confirmed the expression of *Kcnab1* in L6b/SP, similar to that of *Ctgf*. In addition, *Kcnab1* was also observed in other layers in a scattered manner (Figures 2C,D). As we found that its expression was specific to SP in the embryonic stages (see below), we focused on the analysis of *Kcnab1* in this article.

### L6b/SP Association Neurons Expressed *Kcnab1*

From our retrograde tracing experiments and microarray gene analysis, we were inclined to hypothesize that association neurons in L6b/SP were molecularly distinctive. Therefore, brains that were injected with retrograde tracer were processed for ISHH and immunohistochemistry to confirm this hypothesis.

**Figure 3:**
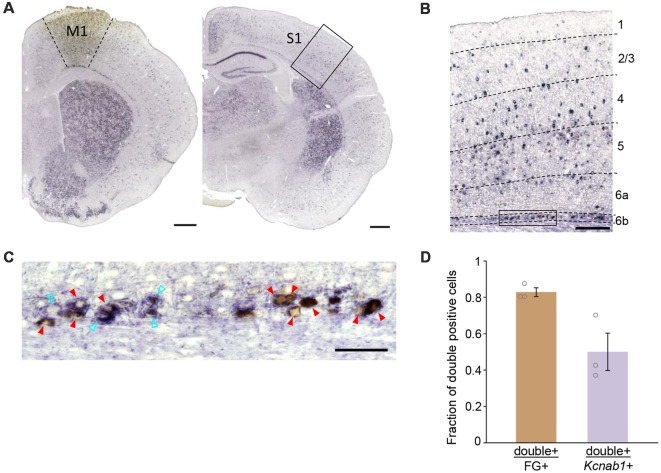
Association neurons in L6b/SP expressed *Kcnab1*. **(A)** ISHH was carried out in combination with retrograde tracing detected by anti-FG antibody to determine *Kcnab1* expression of the association neurons in the L6b/SP of PW3 mouse. The left panel shows FG injection site in M1, revealed by anti-FG staining (brown patch) and the right panel shows the retrogradely labeled region in S1. Scale bars, 500 μm. **(B)** Neurons that were double-positive for FG (brown) and *Kcnab1* (violet) were observed in L6b as well as the upper layers in S1. Dashed lines indicate borders of cortical layers. Scale bar, 200 μm. **(C)** Higher magnification view of the boxed area in **(B)**. Red arrowheads indicate double-positive, i.e., association neurons that expressed *Kcnab1*. Cyan open arrowheads indicate *Kcnab1*-positive neurons that were not retrogradely labeled. Scale bar, 50 μm. **(D)** Summary data showing the overlap between neurons retrogradely labeled from M1 (FG+) and *Kcnab1*+ neurons in L6b/SP at PW3 (*n* = 366 FG+ neurons and *n* = 634 *Kcnab1*+ neurons from *N* = 3 mice). See also Supplementary Table S1 for the details of quantification results.

From our retrograde tracing experiment in combination with ISHH, we observed M1-projecting neurons in L6b/SP in S1 of PW3 mice that were double-positive for FG (detected by anti-FG antibody) and *Kcnab1* (Figures 3A–C). We examined a total of 366 neurons in S1 L6b/SP (*N* = 3 mice) that were retrogradely labeled and 83.0 ± 0.02% [mean ± standard error of the mean (SEM)] of them expressed *Kcnab1* (Figure 3D, Supplementary Figure S1 and Supplementary Table S1). On the other hand, among 634 *Kcnab1*-positive neurons in L6b/SP, 50.1 ± 0.10% were of association type. We did not observe any obvious regional biases in cellular distributions of FG+ and *Kcnab1*+ neurons within S1 (Supplementary Figure S1). Similarly, at the neonatal stage (injection at PD2 and brains fixed at PD4), a proportion of association neurons in L6b/SP expressed *Kcnab1* (data not shown).

### *Kcnab1* Expression During Embryonic Stage Was Restricted to L6b/SP

**Figure 4:**
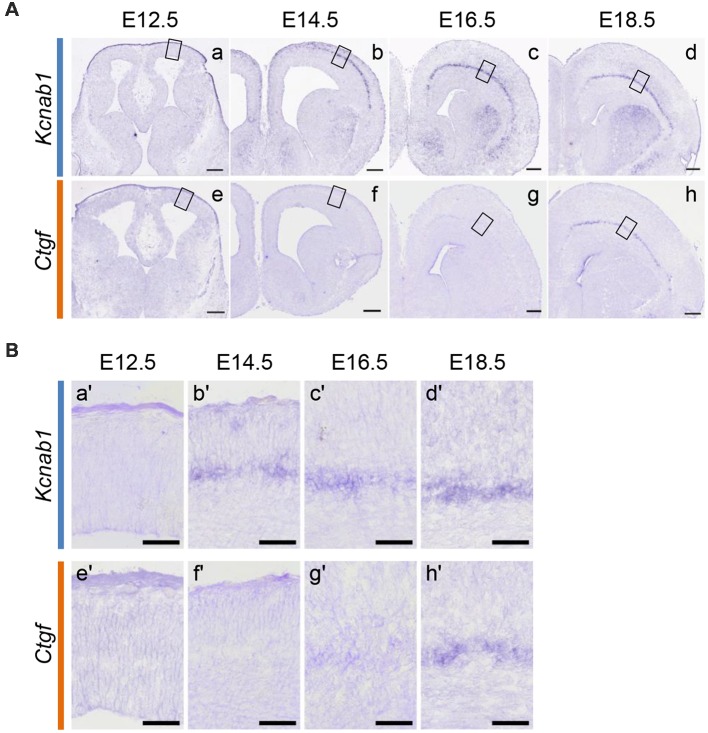
*Kcnab1* expression in developing mouse cortex was detected as early as E14.5. **(A)** ISHH for *Kcnab1* **(a–d)** and *Ctgf* **(e–h)** was carried out on coronal sections of the mouse brain at different embryonic stages (E12.5, E14.5, E16.5, and E18.5). *Kcnab1* expression was observed starting at E14.5 and it began earlier when compared to *Ctgf* expression. Scale bar, 200 mm (micrometer). **(B)** Higher magnification of boxed regions in **(A)**. *Kcnab1* expression was restricted to the SP during the embryonic stage **(b′–d′)**. Scale bar 50 μm.

Since L6b/SP neurons with a unilateral projection expressed *Kcnab1* at both the neonatal and postnatal stages, we set out to examine the onset of *Kcnab1* expression in the mouse cortex and whether the expression pattern was maintained throughout development. First, we carried out ISHH for *Kcnab1* on coronal sections of the mouse brain at different embryonic stages. *Kcnab1* expression was observed in the SP as early as E14.5 (Figure 4), shortly after the SP was formed. Interestingly, *Kcnab1* expression was consistently restricted to the SP, the earliest-formed layer, and not detected in other layers throughout embryonic development. *Kcnab1* has an earlier onset in comparison to the established L6b/SP marker *Ctgf*, of which the earliest expression was detected at E16.5 among the stages tested.

### *Kcnab1* Expression Spread to Other Layers in the Postnatal Stages

**Figure 5:**
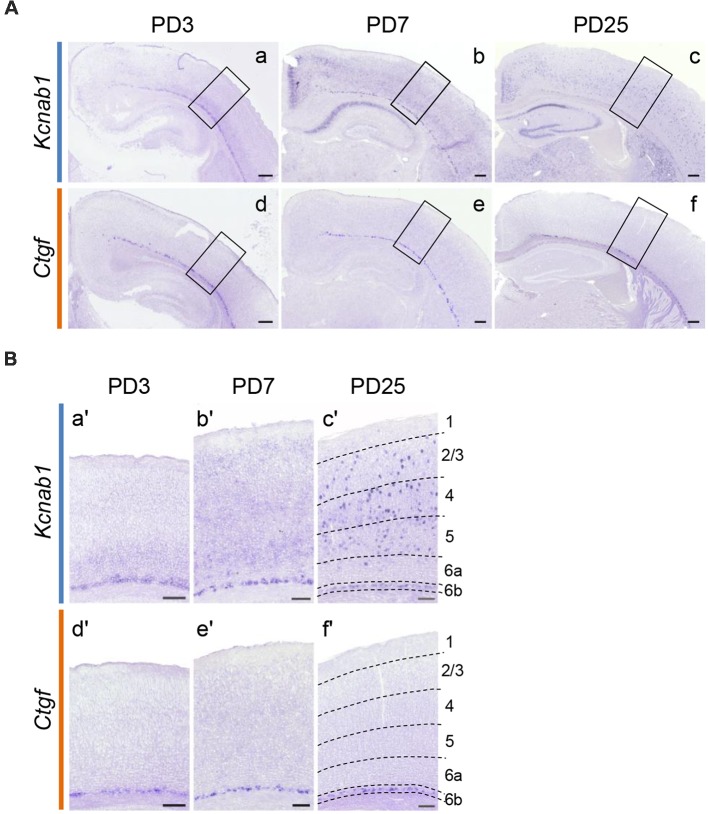
*Kcnab1* expression pattern changed during postnatal development. **(A)** ISHH for *Kcnab1* was carried out on coronal sections of the mouse brain at selected postnatal stages (PD3, PD7, and PD25). Scale bars, 200 μm. **(B)** Higher magnification view of boxed regions in **(A)**. Weak *Kcnab1* expression was detected in deep layers above L6b at PD3 **(a,a′)**. *Kcnab1* expression gradually spread to the more superficial layers and some cells with higher expression appeared **(b,b′)**, becoming evidently scattered in the upper layers at PD25 **(c,c′)**. In comparison, *Ctgf* was limited to L6b throughout postnatal development (**d–f**; **d′–f′**). Dashed lines in **(c′)** and **(f′)** indicate borders of cortical layers. Scale bars, 100 μm.

Our results demonstrated that *Kcnab1* expression in the mouse cortex changes as the brain matures. *Kcnab1*-positive neurons were constrained to L6b/SP during embryonic age (Figure 4). However, the expression was less localized in the PD21 brain (Figure 2C). We went on to analyze the expression pattern during early postnatal stages in order to clarify when exactly the expression in other layers started. Our comparison ISHH results showed that *Kcnab1* expression was apparent in the upper layers at stage PD3 onwards (Figures 5A,B). As the cortical layers became more prominent, *Kcnab1* expression in L6b/SP became weaker whereas numbers of *Kcnab1*-expressing neurons in other layers increased. *Kcnab1*-positive neurons in the upper layers were sparse and scattered in layers 2/3, 4, and 5, seemingly with no specific organization pattern (Figure 5B). Together with the expression during the embryonic stage, these data showed that *Kcnab1* is expressed in a bipartite manner: a stable expression in the L6b/SP throughout the cortical development and a dynamic expression in other layers that appears postnatally.

### *Kcnab1* Neurons Were a Distinct Subpopulation in L6b/SP

**Figure 6:**
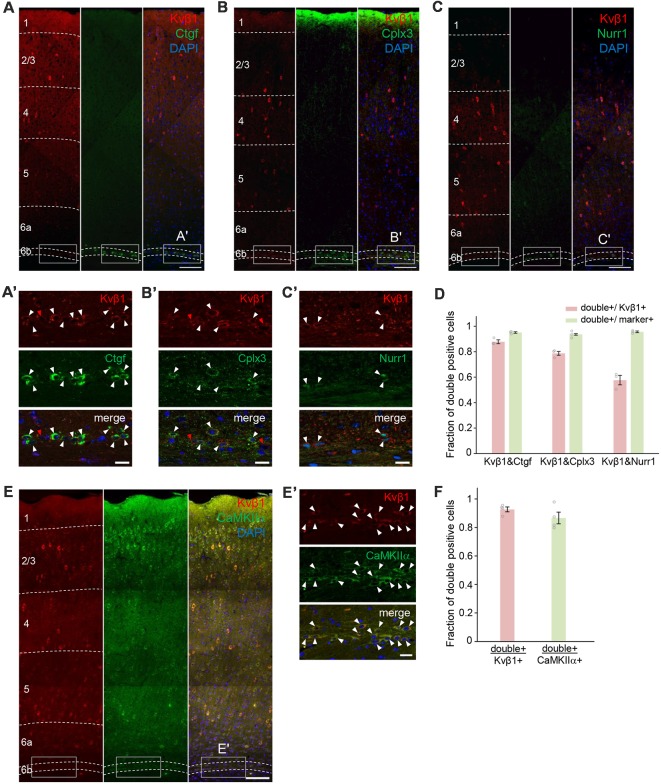
Cellular co-localization of the protein product Kvβ1 and L6b/SP markers in the adult S1. Double immunostaining experiments revealed co-expression of Kvβ1 and L6b/SP markers **(A)** Ctgf, **(B)** Cplx3 and **(C)** Nurr1 in L6b neurons. Dashed lines indicate borders of cortical layers. Scale bars, 100 μm. Boxed areas in **(A–C)** are shown in higher magnification in **(A′–C′)**. White arrowheads indicate double-positive L6b neurons. Red arrowheads indicate L6b neurons that expressed only Kvβ1. Scale bars, 20 μm. **(D)** Summary data showing degrees of co-localization. The bar diagrams illustrate the fraction of double-positive cells to Kvβ1- (red) and marker- (green) positive L6b cells. Staining combinations are shown at the bottom of the bars. Kvβ1-positive neurons in L6b expressed all three markers but the proportion of overlap varied. Error bars represent standard error of the mean (SEM; *N* = 3 animals per marker group). See also Supplementary Table S2 for the details of quantification results. **(E)** Double immunostaining with excitatory neuronal marker CaMKIIα showed that the majority of Kvβ1-expressing neurons were also CamKIIα-immunoreactive. Dashed lines indicate borders of cortical layers. Scale bar, 100 μm. **(E′)** Higher magnification images of boxed areas in **(E)** and arrowheads indicate Kvβ1-CaMKIIα co-localization. Scale bars, 20 μm.** (F)** Summary data showing high percentages of double-positive neurons both in Kvβ1-expressing neurons and in CaMKIIα-expressing neurons in L6b. Error bars represent SEM (*N* = 4 animals). See also Supplementary Table S2 for the details of quantification results.

*Kcnab1* expression was observed in the mouse cortex shortly after the formation of the SP and it remained throughout development. As a step to further characterize this neuronal subclass in the L6b/SP, we tested the co-localization extent of protein product Kvβ1 and selected L6b/SP markers Ctgf, Cplx3, and Nurr1 (Figures 6A–C) in adult brains. All three markers label neurons in a thin band that comprises 2–3 rows of densely packed cells directly above the white matter, with a certain variation in different cortical areas (Liu and Baker, 1999; Arimatsu et al., 2003; Hoerder-Suabedissen et al., 2009; Wang et al., 2011). L6b/SP neurons identified by these markers are known to overlap to a varying degree (Hoerder-Suabedissen et al., 2009, 2013). We examined a total of 409 Kvβ1-immunoreactive neurons in L6b/SP (in three animals) and a majority (87.8 ± 0.02%) of them co-expressed Ctgf. Among 414 Kvβ1-immunoreactive neurons in L6b/SP (in three animals), more than three quarters (78.7 ± 0.02%) were Cplx3-positive, whereas 57.7 ± 0.04% of a total of 462 Kvβ1-positive neurons examined expressed Nurr1 (Figure 6D and Supplementary Table S2). Among the marker-expressing neurons, the vast majority of them (93.6–95.6%) were Kvβ1-positive (Figure 6D and Supplementary Table S2). Thus, *Kcnab1*-expressing neurons constitute a distinct subpopulation of L6b/SP neurons.

To gain insight into the neurochemical characteristics of *Kcnab1*-expressing neurons, we also analyzed the co-localization of Kvβ1 and CaMKIIα, a general marker for excitatory neurons in the cortex (Benson et al., 1992; Jones et al., 1994; Zhang et al., 2006; Wang et al., 2013; Pinto and Dan, 2015). Among 837 Kvβ1-positive neurons in L6b/SP, 92.7 ± 0.02% were CaMKIIα-positive (Figures 6E,F and Supplementary Table S2), suggesting that the majority of *Kcnab1*-expressing neurons were excitatory. A recent study reported that no GABAergic neuron was detected among *Ctgf*-expressing neurons (Boon et al., 2018). As approximately 12% of *Kcnab1*-expressing neurons were *Ctgf*-negative (Figure 6D and Supplementary Table S2), we examined single cell RNAseq data of cortical neurons (Tasic et al., 2016) to see if *Kcnab1* is expressed in GABAergic neurons in L6b. Among 41 neurons dissected from L6b, *Kcnab1* transcripts were detected in 50.0% of GABAergic neurons as well as in 90.9% of glutamatergic neurons (Supplementary Figure S2). Notably, analysis on the RNAseq data further showed that *Kcnab1*-expressing neurons in other layers contained both glutamatergic and GABAergic neurons (Supplementary Figure S2). This was also confirmed by the analysis on another RNAseq data set by Zeisel et al. (2015; Supplementary Figure S3).

### All Cortical Layers Expressed a Single *Kcnab1* Splice Variant

**Figure 7:**
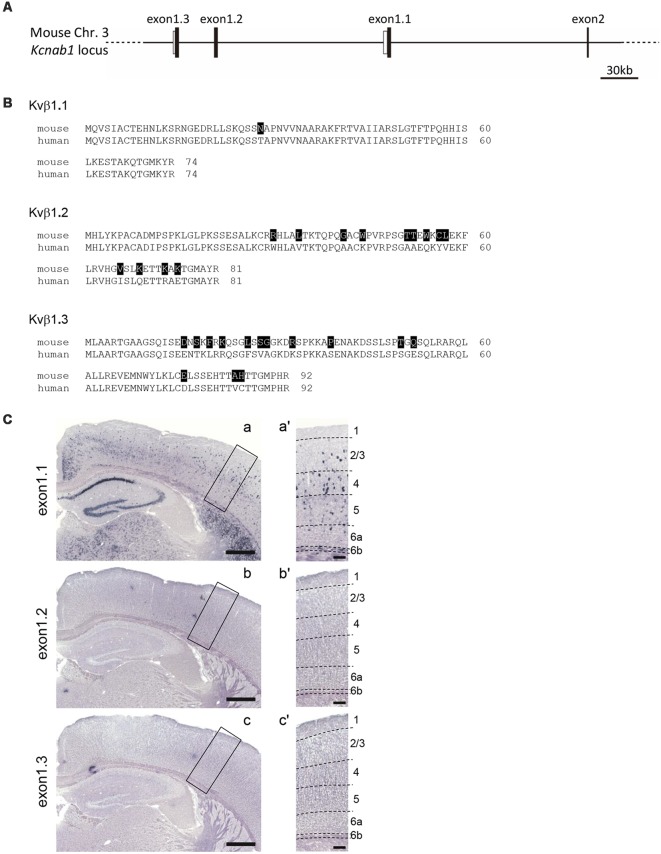
*Kcnab1* splice variant *Kcnab1.1* was expressed in the mouse cortex. **(A)** Genomic structure of 5′ part of the *Kcnab1* gene locus showing three alternative first exons (exons 1.1–1.3). Filled and open bars indicate coding and non-coding part of the exons. **(B)** Amino acid sequence alignments of the three alternative N-terminals of the mouse (m) and human (h) Kvβ1 protein encoded by three alternative first exons. The residues in mKvβ1 different from those in hKvβ1 are shown in white letters on black background. **(C)** ISHH for *Kcnab1* splice variants was carried out on PD25 mouse brain sections using the probes specific for exons 1.1–1.3. Only *Kcnab1.1* expression was observed in the cortex **(a)**. Neither *Kcnab1.2* nor *Kcnab1.3* was present in any layers of any cortical areas **(b,c)**. Higher magnification of boxed regions in **(a–c)** is shown in **(a′–c′)**, an area in S1. Dashed lines indicate borders of cortical layers. Scale bars, 500 μm **(a–c)**; 100 μm **(a′–c′)**.

As the presence of three alternative first exons (exon1.1, 1.2 and 1.3) was reported for the human orthologue of *Kcnab1* (England et al., 1995; McCormack et al., 1995) and our probe for mouse *Kcnab1* spanned from the last quarter of the coding sequence to 3′ UTR shared among three variants, we examined which variant was expressed in the cortex. In the Ensembl genomic database, neither exon 1.2 nor 1.3 was annotated. Thus, we performed the homology search using human orthologous exons as queries and found corresponding mouse exons 1.2 and 1.3 at 132 kb and 169 kb upstream of the exon 1.1, respectively (Figures 7A,B). Amino acid identities between human and mouse proteins within N-terminals encoded by the first exons were 98.6%, 84.0%, and 84.8% for Kvβ1.1, 1.2, and 1.3, respectively. ISHH with the probes specific to each exon revealed that only exon 1.1 among the three variants was expressed in the PD25 cerebral cortex (Figure 7C), recapturing the staining pattern with the probe for 3′ part of the gene (Figure 2C). This held true also for early postnatal stage PD3 (data not shown). Therefore, the expression pattern of *Kcnab1* described in this study was explained by that of the variant 1.1 alone.

## Discussion

The main findings of this study are: (1) that a discrete population of L6b/SP neurons project unilaterally from S1 to M1, and no callosal projection was observed; (2) that *Kcnab1* identifies a proportion of this subclass of L6b/SP neurons with an inter-areal projection; (3) that *Kcnab1* is co-expressed with known L6b/SP markers Ctgf, Cplx3, and Nurr1 to different degrees at postnatal stage; and (4) that *Kcnab1* expression is restricted to L6b/SP during the early developmental stage but scattered *Kcnab1*-positive neurons were observed in the upper layers of postnatal cortices.

L6b/SP neurons are strategically located at the interface of the developing cortex and they also occupy a pivotal time window during which cortical connections begin to establish. Thus, axon projections that originate from this region are most likely to influence subsequent functional connectivity organization as cortical lamination proceeds. Projection from L6b/SP as a whole is commonly categorized as corticocortical and corticothalamic, both of which involve morphologically diverse cells (Zhang and Deschênes, 1997; Kumar and Ohana, 2008; Briggs, 2010; Thomson, 2010; Pichon et al., 2012; Marx and Feldmeyer, 2013; Vélez-Fort et al., 2014; Hoerder-Suabedissen et al., 2018). It is of broad opinion that L6b/SP neurons in rodents send few collaterals to the contralateral cortex (De Carlos and O’Leary, 1992; Ozaki and Wahlsten, 1998; Del Río et al., 2000; Hoerder-Suabedissen and Molnár, 2012). A recent report using a transgenic mouse line showed callosal projections from L6b/SP neurons in P7 mice (Hoerder-Suabedissen et al., 2018), although it seems rather hard to exclude the possibility that these projections were from a small population of L6a neurons found in this particular line (Drd1a-Cre (FK164) at GENSAT^5^). In this study, by retrograde tracing, we observed only ipsilateral inter-areal projection from L6b/SP neurons, and this was consistent at different developmental stages. However, we are yet to confirm if these unilaterally projecting-L6b/SP neurons in S1 solely project to M1 since L6b/SP neurons were reported to have multiple long-range targets (Hoerder-Suabedissen and Molnár, 2012; Hoerder-Suabedissen et al., 2018). Moreover, neonatal SP neurons were shown to project to the opposite hemisphere in other mammalian species (Chun et al., 1987; Antonini and Shatz, 1990). Our finding that *Kcnab1* identifies a proportion of the ipsilaterally-projecting neurons in L6b/SP does not exclude the possibility that there are, although most likely minor, SP neurons that have contralateral projections that were not identified in this study due to experimental limitations such as tracer injections into a very restricted area compared with broad applications into the white matter or the corpus callosum in other studies (Ozaki and Wahlsten, 1998; Del Río et al., 2000; Hoerder-Suabedissen and Molnár, 2012; Boon et al., 2018). Hence, it would be most ideal if the tracing experiment is repeated with transgenic reporter line that would allow further characterization of the association neuron subclass, the axon trajectory that originates from this area, and their targets that may include other cortical regions.

Previous studies that combined birth dating of SP neurons and co-expression of SP markers concluded that Ctgf, Cplx3, and Nurr1 indeed labeled early-born SP neurons and, more importantly, a considerable proportion of these early-born neurons survived after the formation of the cortical plate (Liu and Baker, 1999; Arimatsu et al., 2003; Heuer et al., 2003; Hoerder-Suabedissen et al., 2009; Hoerder-Suabedissen and Molnár, 2013). In the adult cortex, we found a prominent overlap between *Kcnab1* protein product Kvβ1-immunoreactive neurons and Ctgf, Cplx3, and Nurr1, to varying degrees. This indicates that a considerable number of L6b/SP neurons of distinct identity that were generated during the embryonic stage remain into adulthood. Moreover, our data suggest that there is a molecular subdivision of L6b/SP association neurons. It is possible that one or more of the markers tested are expressed in the *Kcnab1*-negative population (17%) of the M1-projecting L6b/SP neurons in S1. On the other hand, *Kcnab1*-expressing neurons without projection to M1 may consist of neurons projecting to other cortical areas including S2, or even to subcortical targets. Furthermore, there is a subdivision of *Kcnab1*-expressing neurons in terms of neurotransmitters, since *Kcnab1* expression in 50% of GABAergic neurons in L6B/SP was evidenced in the analysis on the published single cell RNAseq data sets (Supplementary Figures S2, S3). A similar analysis revealed that some GABAergic neurons expressed the *Camk2a* gene (Supplementary Figure S4), implying that some of the Kvβ1/CaMKIIα double-positive neurons we detected were possibly GABAergic neurons. It would be an intriguing future work to examine if GABAergic *Kcnab1*-expressing neurons in L6b/SP have long-range ipsilateral corticocortical projections as shown for a small proportion of GAD65-expressing L6b/SP neurons (Boon et al., 2018).

Kvβ1 is an intracellular regulatory subunit of *shaker*-type Kv1 channel that rapidly blocks the potassium influx by binding to the channel pore with its N-terminal “ball” domain (Sewing et al., 1996). This fast transient (A-type) current enables neurons to fire repetitively in an input intensity-dependent manner (Connor and Stevens, 1971). The A-type current and input-dependent increase of firing rates are reported for SP neurons, but not for immature pyramidal neurons in the cortical plate of neonatal rat cortex (Luhmann et al., 2000), which is consistent with Kvβ1 expression in L6b/SP. Furthermore, enriched expression in human embryonic SP was reported (Miller et al., 2014), suggesting the conserved regulation and functions of Kvβ1 in prenatal SP between primates and rodents. Previous molecular profiling has reported a variety of genes that are SP-specific at certain developmental stages and only a small number of specific genes—Nurr1, Ctgf, Cplx3, Nxph4, Inpp4b, Htr1d and Tpd52I1 are present in L6b/SP throughout development (Hoerder-Suabedissen et al., 2013). Interestingly, we observed Kvβ1 expression in other cortical layers besides L6b/SP only in the postnatal mouse brain. Thus the unresolved question is whether these Kvβ1 neurons that were scattered in the upper layers were of SP origin (Ozair et al., 2018) or if they were of newly acquired expression-type neurons as development progresses. This would be essential to clarify the function of this L6b/SP subpopulation at different developmental stages since Kvβ1 has been implicated to be involved in certain types of learning and memory (Giese et al., 1998; Murphy et al., 2004).

Heterogeneity of L6b/SP neurons remains a great hurdle to understand the enigmatic role of this likely transient structure. L6b/SP is a robust zone that contains both inhibitory and excitatory neurons (Hoerder-Suabedissen and Molnár, 2015), and it also forms subcortical connections (Killackey and Sherman, 2003; Roth et al., 2016; Chevée et al., 2018; Hoerder-Suabedissen et al., 2018). Although connection between L6b/SP within and outside of the cortex seems to vary across mammalian species (De Carlos and O’Leary, 1992; Ozaki and Wahlsten, 1998; Del Río et al., 2000; Briggs and Usrey, 2007), it is important to note that L6b/SP neurons are essentially involved in cortical information processing. Previous reports have suggested that neurons in L6b/SP go through timely functional changes throughout development (Friedlander and Torres-Reveron, 2009). In this study, however, we have identified a group of L6b/SP association neurons that expressed a specific splice variant of *Kcnab1*, and the expression in L6b/SP was maintained at all developmental stages. This molecular authenticity that spans development could be a plausible mechanism that modulates L6b/SP neuron specification during cortical maturation, and this would add to the increasing efforts to characterize L6b/SP neurons, thus elucidating the role of L6b/SP neurons in SP development, axon extension, and subsequent brain network establishment.

## Ethics Statement

All animal experiments were approved by Animal Research Committee of University of Fukui and Animal Experimentation Committee of Osaka University, and performed in accordance with Regulations for Animal Research at University of Fukui and Regulations on Animal Experimentation at Osaka University.

## Author Contributions

ST, YO, and MS designed the research. ST, YO, TS, MT, and MD performed the experiments. ST, YO, TS, and HA analyzed the data. ST, YO, and MS wrote the manuscript.

## Conflict of Interest Statement

The authors declare that the research was conducted in the absence of any commercial or financial relationships that could be construed as a potential conflict of interest.

## Footnotes

^1^http://www.ensembl.org/

^2^https://www.ebi.ac.uk/Tools/psa/genewise/

^3^www.ncbi.nlm.nih.gov/geo

^4^http://linnarssonlab.org/cortex

^5^http://www.gensat.org/
