# Supplementary material for: Kcnab1 Is Expressed in Subplate Neurons With Unilateral Long-Range Inter-Areal Projections
Source: Front Neuroanat. 2019 May 3;13:39. doi: 10.3389/fnana.2019.00039 (PMC6509479; doi:10.3389/fnana.2019.00039)
Supplement: Supplementary file 1 [file Image_1.pdf]

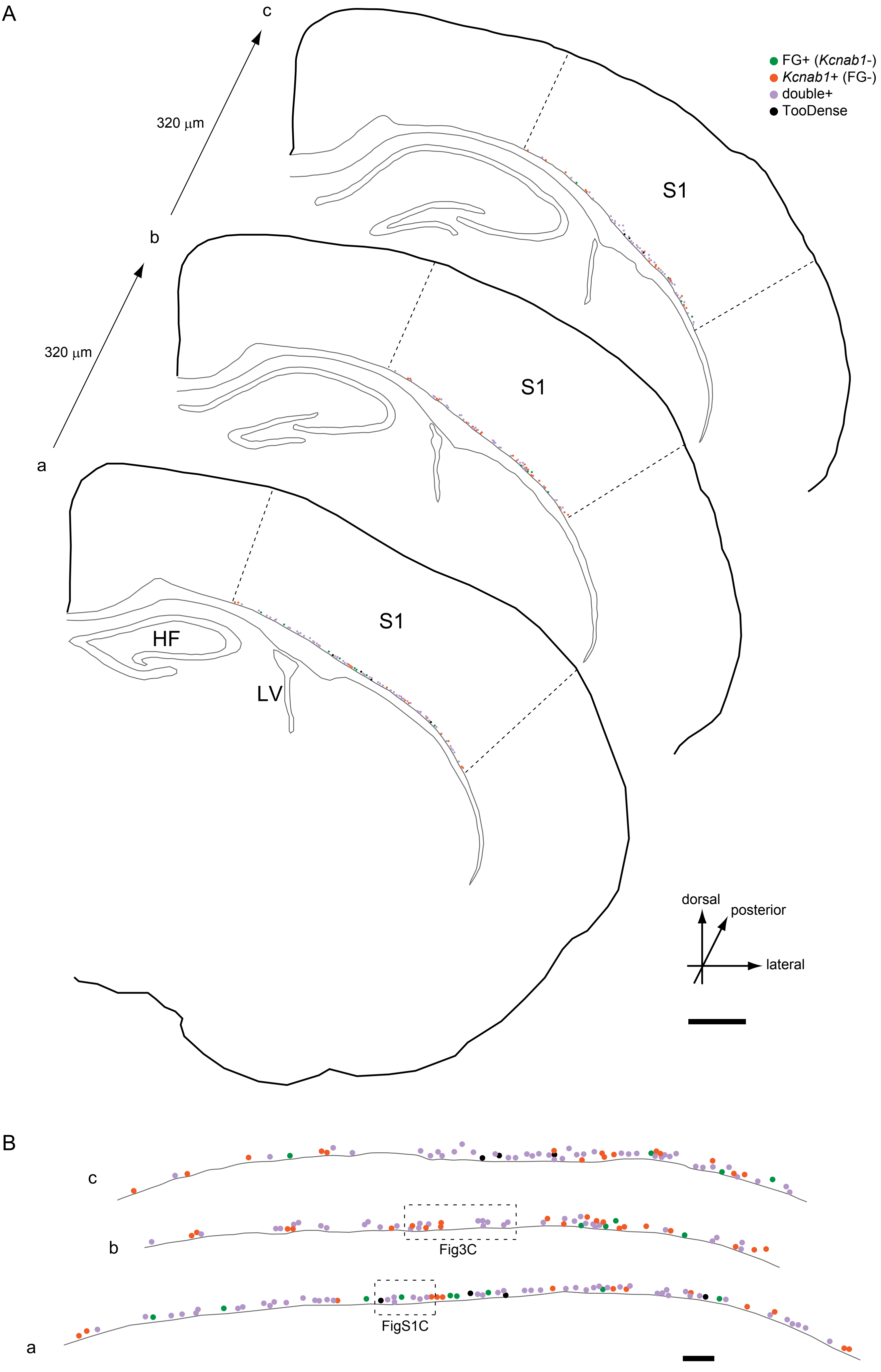

A
B
a
b
c
320 µm
320 µm
FG+ (Kcnab1-)
Kcnab1+ (FG-)
double+
TooDense
S1
S1
S1
HF
LV
dorsal
posterior
lateral
Fig3C
FigS1C

C

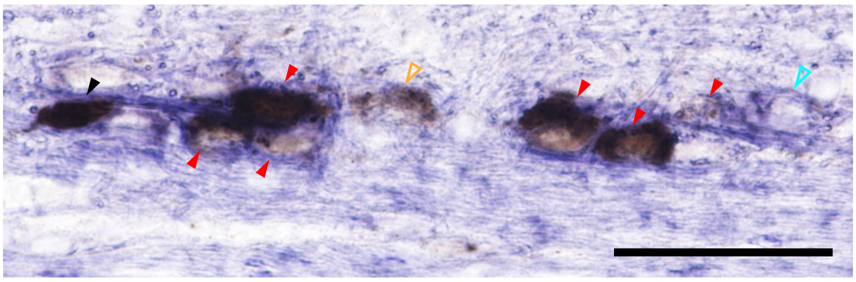

Supplementary Figure S1. A distribution map of FG+, *Kcnab1*+ and double+ cells in L6b/SP in S1. (**A**) Filled circles color-coded for stainings were placed in the positions of respective cells on the traces of three sections analyzed for animal #1 (**a**-**c**. See Supplementary Table S1 for cell counts). Color coding different from Figure 3D or Supplementary Table S1 is used for better visualization and color distinction of circles. Only cells in S1 were counted and mapped. TooDense cells were excluded from the mean calculation. The order of and the distance between the sections are shown on the left. HF, hippocampal formation. LV, lateral ventricle. Scale bar, 500 μm. (**B**) Enlarged views of L6b/SP in S1 of each map shown in (A). The maps were rotated to align roughly to the horizontal line. No obvious distribution bias for each cell type was detected. Scale bar, 100 μm. (**C**) An example image that shows association neurons in L6b neurons that expressed *Kcnab1*. Red arrowheads indicate double positive, i.e. association neurons that expressed *Kcnab1*. The Cyan open arrowhead indicate *Kcnab1*-positive neurons that were not retrogradely labeled. The yellow open arrowhead indicates a retrogradely-labeled L6b neuron that was not *Kcnab1*-positive. The black arrowhead indicates a cell that was stained too densely and was excluded from quantification. Scale bar, 50 μm.
