# Supplementary material for: Kcnab1 Is Expressed in Subplate Neurons With Unilateral Long-Range Inter-Areal Projections
Source: Front Neuroanat. 2019 May 3;13:39. doi: 10.3389/fnana.2019.00039 (PMC6509479; doi:10.3389/fnana.2019.00039)
Supplement: Supplementary file 2 [file Image_2.pdf]

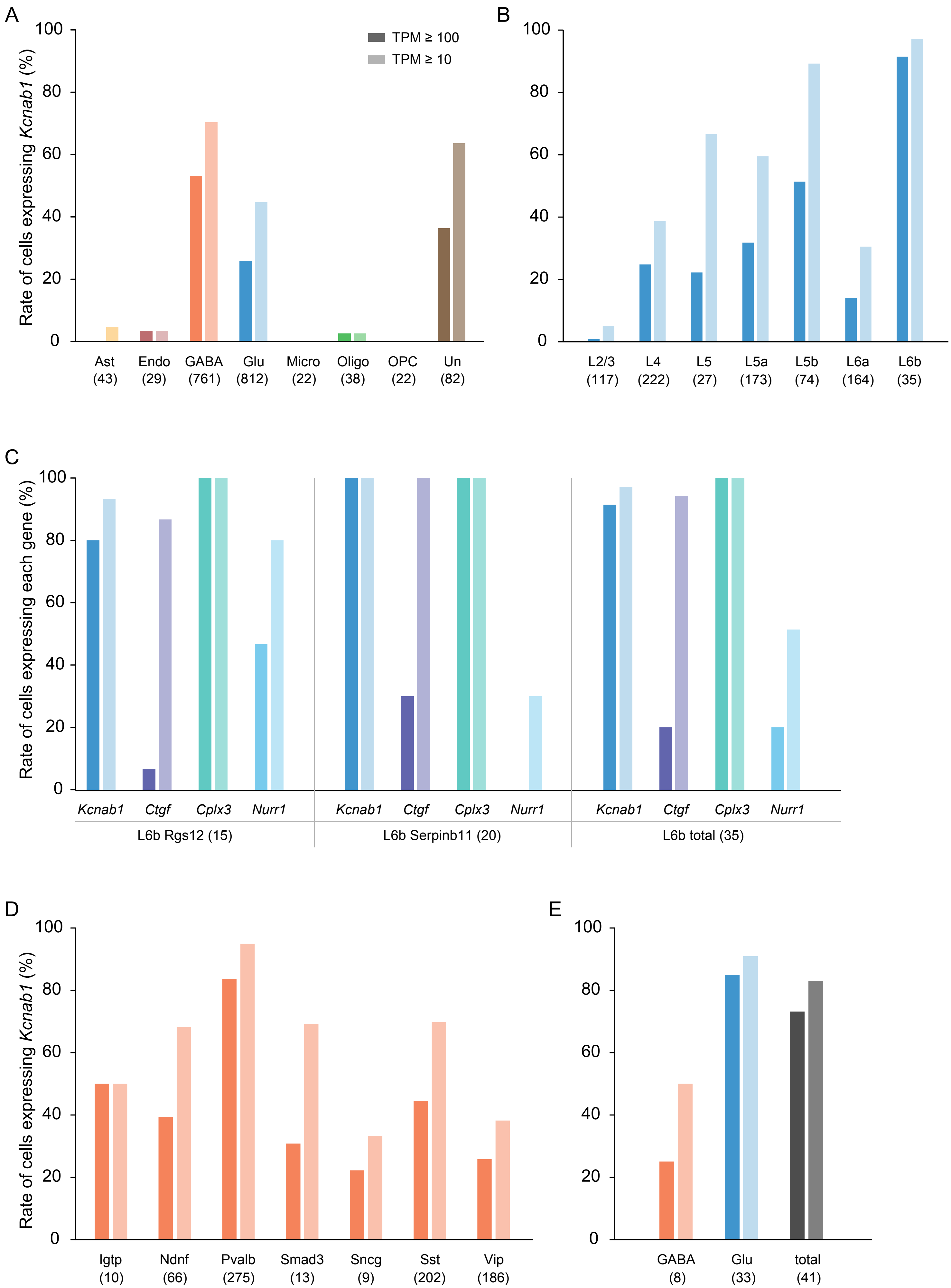
A
B
C
D
E
TPM ≥ 100
TPM ≥ 10
Rate of cells expressing Kcnab1 (%)
Rate of cells expressing each gene (%)
100
80
60
40
20
0
Ast (43)
Endo (29)
GABA (761)
Glu (812)
Micro (22)
Oligo (38)
OPC (22)
Un (82)
L2/3 (117)
L4 (222)
L5 (27)
L5a (173)
L5b (74)
L6a (164)
L6b (35)
Kcnab1
Ctgf
Cplx3
Nurr1
L6b Rgs12 (15)
L6b Serpinb11 (20)
L6b total (35)
Igtp (10)
Ndnf (66)
Pvalb (275)
Smad3 (13)
Sncg (9)
Sst (202)
Vip (186)
GABA (8)
Glu (33)
total (41)

Supplementary Figure S2. Summary of analyses on single cell RNAseq data by Tasic et al., 2016. Darker bars represent the rate of cells that expressed the gene of interest at the level of TPM (transcripts per million) ≥ 100, while light bars are TPM ≥ 10. The number of cells contained in each category (cell type) is shown in parentheses at bottom of the charts. (**A**) A bar chart of the rate of *Kcnab1*-expressing cells in 8 broad types of cells. Ast, astrocytes; Endo, endothelial cells; GABA, GABAergic interneurons; Glu, glutamatergic neurons; Micro, microglia; Oligo, oligodendrocytes; OPC, oligodendrocyte precursor cells; and Un, unclassified cells. (**B**) A bar chart of the rate of *Kcnab1*-expressing glutamatergic neurons in primary cell types combined for each cortical layer. (**C**) Bar charts of the rate of cells expressing each of 4 L6b/SP genes in 2 types of L6b neurons (Rgs12 and Serpinb11) separately (left and middle) and combined (right). (**D**) A bar chart of the rate of *Kcnab1*-expressing GABAergic neurons in primary cell types combined for each class of interneurons. Igtp, interferon gamma–induced GTPase; Ndnf, neuron-derived neurotrophic factor; Pvalb, parvalbumin; Smad3, SMAD family member 3; Sncg, synuclein gamma; Sst, somatostatin; and Vip, vasoactive intestinal peptide. (**E**) A bar chart of the rate of *Kcnab1*-expressing cells in glutamatergic or GABAergic neurons dissected from L6b. See Tasic et al., 2016 for details of cell type clsassification.
