# Supplementary material for: Kcnab1 Is Expressed in Subplate Neurons With Unilateral Long-Range Inter-Areal Projections
Source: Front Neuroanat. 2019 May 3;13:39. doi: 10.3389/fnana.2019.00039 (PMC6509479; doi:10.3389/fnana.2019.00039)
Supplement: Supplementary file 3 [file Image_3.pdf]

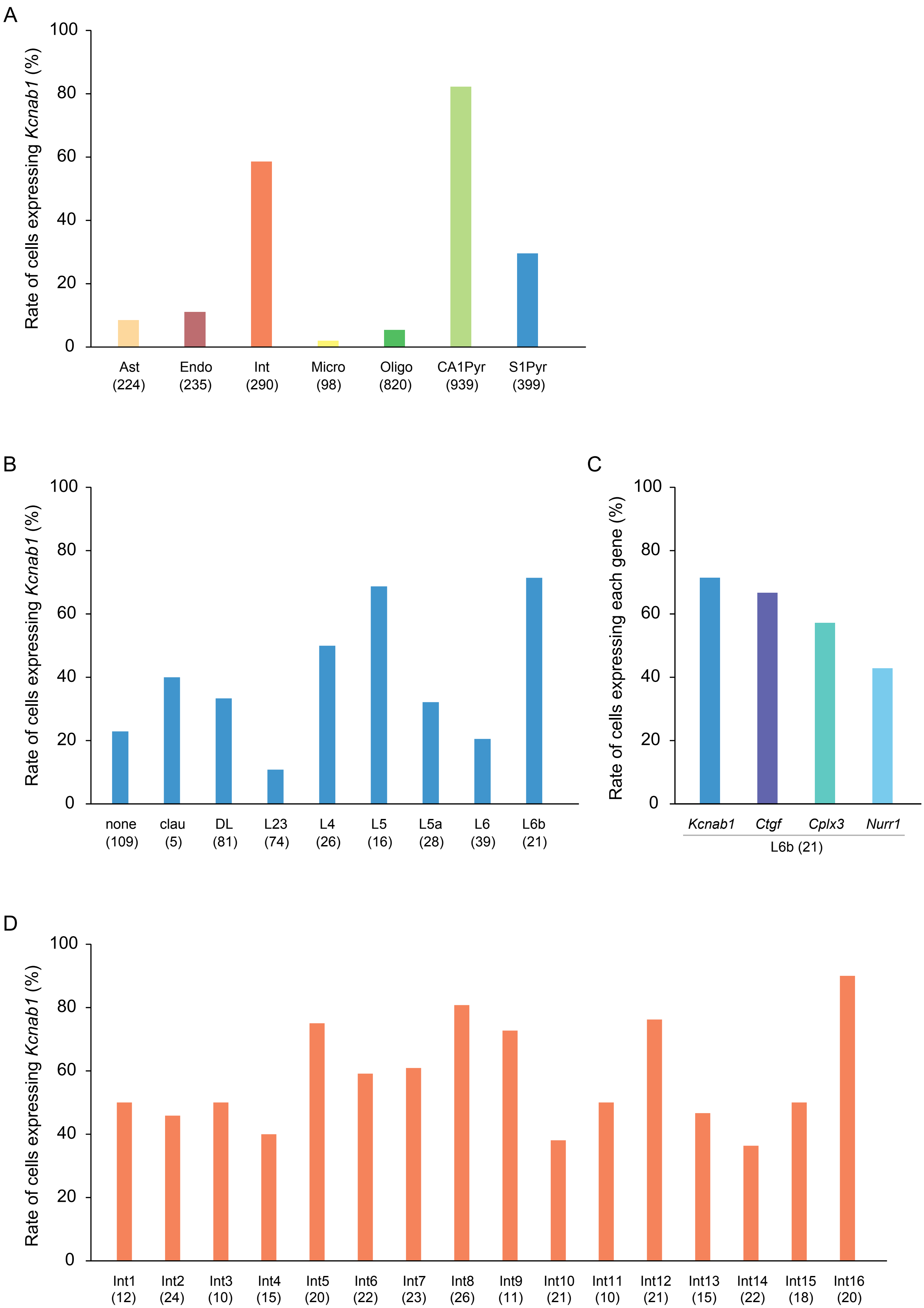
A
Rate of cells expressing Kcnab1 (%)
100
80
60
40
20
0
Ast (224)
Endo (235)
Int (290)
Micro (98)
Oligo (820)
CA1Pyr (939)
S1Pyr (399)
B
Rate of cells expressing Kcnab1 (%)
none (109)
clau (5)
DL (81)
L23 (74)
L4 (26)
L5 (16)
L5a (28)
L6 (39)
L6b (21)
C
Rate of cells expressing each gene (%)
Kcnab1
Ctgf
Cplx3
Nurr1
L6b (21)
D
Rate of cells expressing Kcnab1 (%)
Int1 (12)
Int2 (24)
Int3 (10)
Int4 (15)
Int5 (20)
Int6 (22)
Int7 (23)
Int8 (26)
Int9 (11)
Int10 (21)
Int11 (10)
Int12 (21)
Int13 (15)
Int14 (22)
Int15 (18)
Int16 (20)

Supplementary Figure S3. Summary of analyses on single cell RNAseq data by Zeisel et al., 2015. Bars represent the rate of cells that expressed the gene of interest with the expression score ≥ 1. The number of cells contained in each category (cell type) is shown in parentheses at bottom of each chart. (**A**) A bar chart of the rate of *Kcnab1*-expressing cells in 7 Level-1 classes of cells. Ast, astrocytes + ependymal cells; Endo, endothelial mural cells; Int, interneurons; Micro, microglia; Oligo, oligodendrocytes; CA1Pyr, pyramidal neurons in CA1; and S1Pyr, pyramidal neurons in S1. (**B**) A bar chart of the rate of *Kcnab1*-expressing neurons in Level-2 classes of glutamatergic neurons. clau, claustrum; DL, deep layer. (**C**) A bar chart of the rate of cells expressing each of 4 L6b/SP genes in 21 L6b neurons. (**D**) A bar chart of the rate of *Kcnab1*-expresing neurons in Level-2 classes of interneurons. See Zeisel et al., 2015 for details of cell type classification.
