# Supplementary material for: Kcnab1 Is Expressed in Subplate Neurons With Unilateral Long-Range Inter-Areal Projections
Source: Front Neuroanat. 2019 May 3;13:39. doi: 10.3389/fnana.2019.00039 (PMC6509479; doi:10.3389/fnana.2019.00039)
Supplement: Supplementary file 4 [file Image_4.pdf]

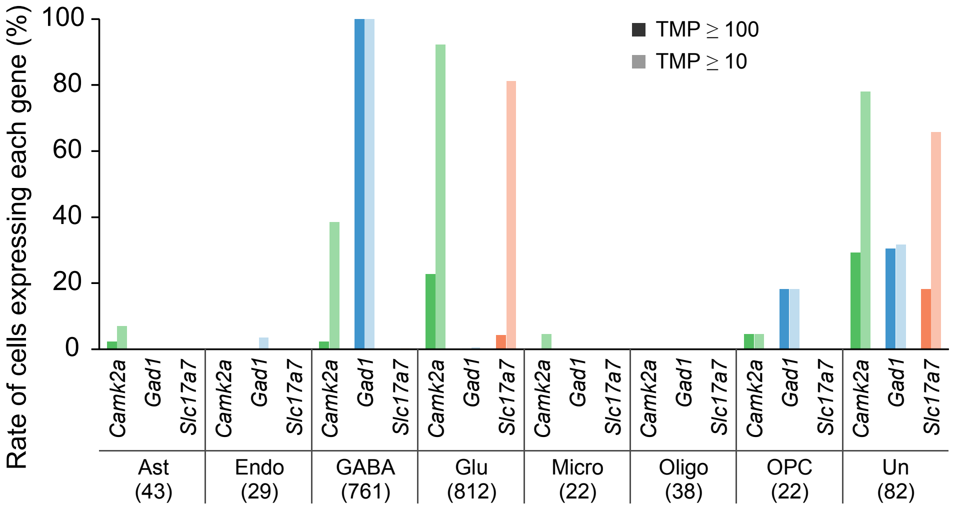

Supplementary Figure S4. Expression profile of the *Camk2a* gene across different cell types in the cortex, in comparison with those of *Gad1* and *Slc17a7* (*Vglut1*) genes.

Bar charts of the rate of cells expressing *Camk2a*, *Gad1*, or *Slc17a7* genes in 8 cell types in the mouse V1. Darker bars represent the rate of cells that expressed the gene of interest at the level of TPM (transcripts per million) ≥ 100, while light bars are TPM ≥ 10. The number of cells contained in each category (cell type) is shown in parentheses at bottom of the charts. Ast, astrocytes; Endo, endothelial cells; GABA, GABAergic interneurons; Glu, glutamatergic neurons; Micro, microglia; Oligo, oligodendrocytes; OPC, oligodendrocyte precursor cells; and Un, unclassified cells. See Tasic et al., 2016 for details of cell type clsassification.
