# Supplementary material for: Kcnab1 Is Expressed in Subplate Neurons With Unilateral Long-Range Inter-Areal Projections
Source: Front Neuroanat. 2019 May 3;13:39. doi: 10.3389/fnana.2019.00039 (PMC6509479; doi:10.3389/fnana.2019.00039)
Supplement: Supplementary file 5 [file Table_1.pdf]

Supplementary Table S1. Quantification results of double labeling with retrograde tracing and ISHH

A. The number of cells counted for each category in each animal.

	# of sections	FG+	*Kcnab1* +	double+	TooDense
animal #1	3	146	182	128	7
animal #2	3	87	164	70	0
animal #3	7	133	288	107	0

B. Fraction of double positive cells in FG+ or *Kcnab1* + cells in each animal. Mean, SD, and SEM for N=3 animals were calculated in 4 different handlings of TooDense cells. The bar graph in Figure 3D was based on exclusion of TooDense cells from calculation, but the differences in the means across the handlings were minute and within the range of SEMs.

TooDense	excluded		counted as FG+		counted as *Kcnab1* +		counted as double+	
	double+ /FG+	double+ /*Kcnab1* +	double+ /FG+	double+ /*Kcnab1* +	double+ /FG+	double+ /*Kcnab1* +	double+ /FG+	double+ /*Kcnab1* +
animal #1	0.877	0.703	0.837	0.703	0.877	0.677	0.882	0.714
animal #2	0.805	0.427	0.805	0.427	0.805	0.427	0.805	0.427
animal #3	0.805	0.372	0.805	0.372	0.805	0.372	0.805	0.372
mean	0.829	0.501	0.815	0.501	0.829	0.492	0.830	0.504
SD	0.042	0.178	0.019	0.178	0.042	0.163	0.045	0.184
SEM	0.024	0.103	0.011	0.103	0.024	0.094	0.026	0.106
