# Supplementary material for: Kcnab1 Is Expressed in Subplate Neurons With Unilateral Long-Range Inter-Areal Projections
Source: Front Neuroanat. 2019 May 3;13:39. doi: 10.3389/fnana.2019.00039 (PMC6509479; doi:10.3389/fnana.2019.00039)
Supplement: Supplementary file 6 [file Table_2.pdf]

Supplementary Table S2. Quantification results of double immunohistochemistry

A. Counting result of the numbers of Kvβ1+, Ctgf+, DAPI+ and double+ cells. Fractions of Kvβ1+ or Ctgf+ cells in DAPI+ cells and those of double+ cells in Kvβ1+ or Ctgf+ cells, as well as their mean, SD, and SEM for N=3 animals were calculated.

	# of sections	Kvβ1+	Ctgf+	DAPI+	double+	Kvβ1+ /DAPI+	Ctgf+ /DAPI+	double+ /Kvβ1+	double+ /Ctgf+
animal #1	3	153	148	339	139	0.451	0.437	0.908	0.939
animal #2	3	131	118	292	113	0.449	0.404	0.863	0.958
animal #3	3	125	113	269	108	0.465	0.420	0.864	0.956
mean						0.455	0.420	0.878	0.951
SD						0.009	0.016	0.026	0.010
SEM						0.005	0.009	0.015	0.006

B. Counting result of the numbers of Kvβ1+, Cplx3+, DAPI+ and double+ cells. Fractions of Kvβ1+ or Cplx3+ cells in DAPI+ cells and those of double+ cells in Kvβ1+ or Cplx3+ cells, as well as their mean, SD, and SEM for N=3 animals were calculated.

	# of sections	Kvβ1+	Cplx3+	DAPI+	double+	Kvβ1+ /DAPI+	Cplx3+ /DAPI+	double+ /Kvβ1+	double+ /Cplx3+
animal #1	3	136	120	320	109	0.425	0.375	0.801	0.908
animal #2	3	117	94	287	88	0.408	0.328	0.752	0.936
animal #3	3	161	135	300	130	0.537	0.450	0.807	0.963
mean						0.456	0.384	0.787	0.936
SD						0.070	0.062	0.030	0.027
SEM						0.040	0.036	0.018	0.016

C. Counting result of the numbers of Kvβ1+, Nurr1+, DAPI+ and double+ cells. Fractions of Kvβ1+ or Nurr1+ cells in DAPI+ cells and those of double+ cells in Kvβ1+ or Nurr1+ cells, as well as their mean, SD, and SEM for N=3 animals were calculated.

	# of sections	Kvβ1+	Nurr1+	DAPI+	double+	Kvβ1+ /DAPI+	Nurr1+ /DAPI+	double+ /Kvβ1+	double+ /Nurr1+
animal #1	3	135	71	283	68	0.477	0.251	0.504	0.958
animal #2	3	139	89	263	84	0.529	0.338	0.604	0.944
animal #3	3	188	121	333	117	0.565	0.363	0.622	0.967
mean						0.523	0.318	0.577	0.956
SD						0.044	0.059	0.064	0.012
SEM						0.025	0.034	0.037	0.007

D. Counting result of the numbers of Kvβ1+, CaMKIIα+, DAPI+ and double+ cells. Fractions of Kvβ1+ or CaMKIIα+ cells in DAPI+ cells and those of double+ cells in Kvβ1+ or CaMKIIα+ cells, as well as their mean, SD, and SEM for N=4 animals were calculated.

	# of sections	Kvβ1+	CaMKIIα+	DAPI+	double+	Kvβ1+ /DAPI+	CaMKIIα+ /DAPI+	double+ /Kvβ1+	double+/ CaMKIIα+
animal #1	2	197	198	349	173	0.564	0.567	0.878	0.874
animal #2	2	184	217	370	173	0.497	0.586	0.940	0.797
animal #3	2	242	236	493	231	0.491	0.479	0.955	0.979
animal #4	2	214	245	501	200	0.427	0.489	0.935	0.816
mean						0.495	0.530	0.927	0.867
SD						0.056	0.054	0.034	0.082
SEM						0.028	0.027	0.017	0.041
